# Practical Microwave-Assisted Recirculated Flow Esterification at Atmospheric Pressure

**DOI:** 10.1021/acsomega.6c03090

**Published:** 2026-06-02

**Authors:** József Schindler, Rebeka Harján, György Keglevich

**Affiliations:** Department of Organic Chemistry and Technology, Faculty of Chemical Technology and Biotechnology, 61810 Budapest University of Technology and Economics, Műegyetem rkp. 3, 1111 Budapest, Hungary

## Abstract

In our previous publications, we reported on esterifications carried out in a circulating flow MW system. It was found that circulation enables direct insight into the reactions from both chemical and energy perspectives. We examined productivity and scalability in terms of substrate, temperature, flow rate, and catalyst, and also analyzed the processes from the point of view of energy. Compared to the conventional linear setup, the circulation resulted in a significantly higher reaction rate, and thus a higher productivity, due to a drastic increase in flow rate. However, it also became clear that higher flow rates impose a substantially higher load on both the MW equipment and the overall system, which may easily lead to pipe perforations or instantaneous seal wear due to the higher operating pressure generated by the back pressure regulator (BPR) control unit. As higher pressures are associated with elevated temperatures, this raises considerable safety concerns. In this article, we aimed to eliminate the BPR unit. While this approach sacrifices a key advantage typically associated with the MW technology by restricting the operation to atmospheric pressure, the capabilities offered by the circulation solution may compensate for the inherent limitations of the new system, and, in turn, the safety risk may be reduced.

## Graphical abstract

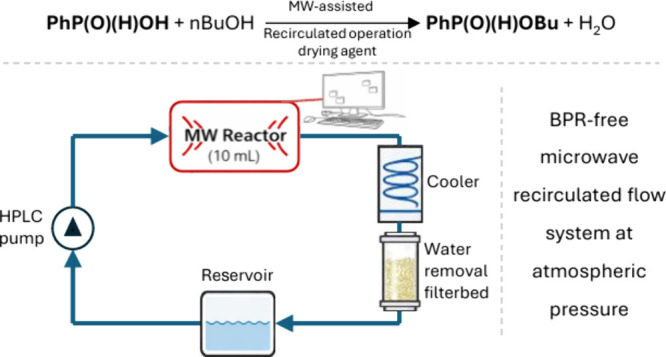

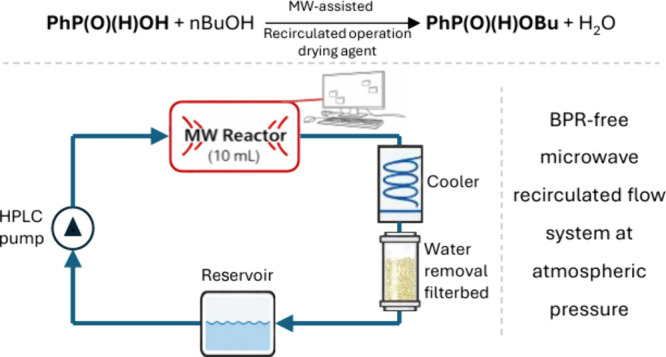

## 1 Introduction

Nowadays, there is a growing demand for technical and technological solutions capable of addressing scale-up challenges.^1–5^ Large-volume chemical processes are predominantly operated under continuous-flow conditions.^6–8^ During the scale-up challenges, developers cannot rely on a batch-oriented approach. Instead, flow-based systems should be sought. There is need to model and gain a deeper understanding of the phenomena that emerge while scaling up.^9–12^ In the context of microwave (MW)-assisted processes, a realistic option may be a simple MW device equipped with a flow reactor. Although MW devices are typically designed for batch reactions, the integration of an inexpensive and straightforward flow cell renders the system suitable for this type of reaction.^13–17^ Beyond the pump, the flow unit comprises a flow cell equipment and a back pressure regulator (BPR). To enable operation above the boiling point, and thus to exploit the well-established advantages of MW reactors, the pump must generate sufficient pressure to prevent evaporation of the reaction mixture at the target temperature. The BPR reduces the elevated pressure in the reactor to 1 bar after the reaction mixture was cooled below its boiling point. Manufacturers offer BPRs with predefined pressures for laboratory and industrial flow systems, ensuring stable pressure control and reproducible reaction conditions.^18–20^ Their operation is typically based on the spring force required via a check valve. However, this technical design inherently limits the applicability in systems containing solid materials, as particles can interfere with the shut-off mechanism and lead to malfunction. To overcome this limitation, the Baxendale group has developed an innovative alternative solution.^21^

It may occur that the pressure in the system increased so rapidly that the BPR was unable to respond adequately, ultimately resulting in an explosion. Fortunately, in these instances, a maximum of 15 mL of reaction mixture was boiled away. As such events had been anticipated and appropriate precautionary measures were introduced, no significant damage occurred. Nevertheless, these events highlight the important message that even with precise preparation and careful operation, the risk of accidental failure cannot be fully eliminated. Our research group further studied the possibilities for developing a system based on our previous recirculation experiments^22^ and the energy background observed.^23^ Our goal was to develop a setup capable of safe operation without reliance on pressure-maintaining components that are difficult to manage.

This article reports on the viability of eliminating the BPR and describes a step-by-step pathway that ultimately led to the successful implementation of this concept. The present work demonstrates that MW-assisted recirculating flow systems can be operated safely at atmospheric pressure without the use of a BPR, while maintaining useful reaction productivity.

## 2 Results and Discussion

### 2.1 Model Reaction

The model reaction selected was the esterification of phenyl-*H*-phosphinic acid (**1**) with butyl alcohol carried out in a solvolytic manner (Scheme 1). The reagent mixture comprised phenyl-*H*-phosphinic acid in butyl alcohol in a n_BuOH_/n_PhP(O)H(OH)_ molar ratio of 15.6:1 meaning a 10% m/V concentration.

**Scheme 1:**
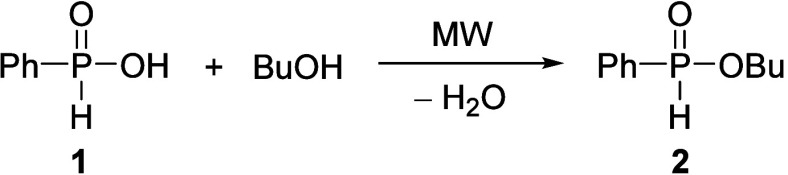
MW-Assisted Direct Esterification of Phenyl-*H*-phosphinic Acid (1) with Butyl Alcohol Chosen as the Model Reaction for the Investigation of the Recirculated Flow Synthesis

### 2.2 MW System without BPR

We previously developed a recirculation-based flow setup, which has now been further modified by eliminating the pressure-maintaining unit (BPR) (Figure 1). The volume of the flow cell (V_f_) was 10 mL.

**Figure 1:**
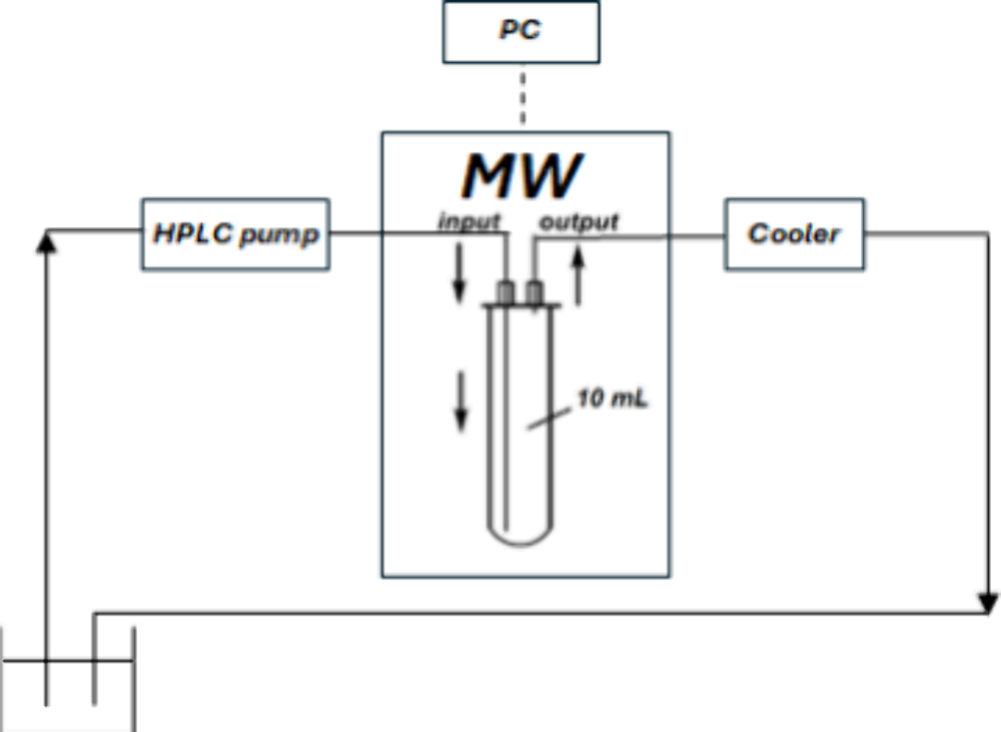
Sketch of the continuous flow system elaborated without BPR.

#### 2.2.1 Setting the Flow Rate and Temperature Parameters

In all experiments, we circulated 25 mL of reaction mixtures containing 5% butyl methylimidazolium hexafluorophosphate [bmim]­[PF6] (IL) as the catalyst. IL, such as [bmim]­[PF6], were selected not only for their catalytic properties, but also as efficient MW absorbers (Microwave-Assisted Ionic Liquid, MAIL technology). These specialized additives facilitate rapid heating and can induce selective energy dissipation at the molecular level.^24^ We varied the flow rates between 10 and 25 mL/min.

In order to further reduce the previously applied temperatures of 160 to 140 °C, we evaluated our results from a different perspective. Previously, we plotted conversion data as a function of time for different flow rates (Figure 2) Here, however, we raised an additional question: for a given flow rate, what conversion values correspond to the temperatures measured at specific time points?

**Figure 2:**
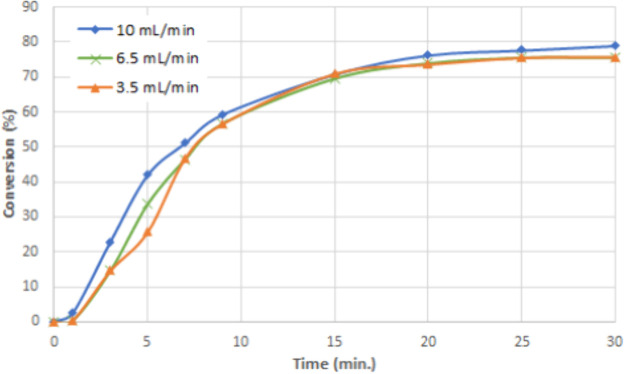
Dependence of the conversion on time at different flow rates.

To answer this, first the time–temperature relationship at various flow rates was examined (Figure 3). Then, using these data, we plotted the conversion values as a function of the corresponding temperatures (Figure 4).

**Figure 3:**
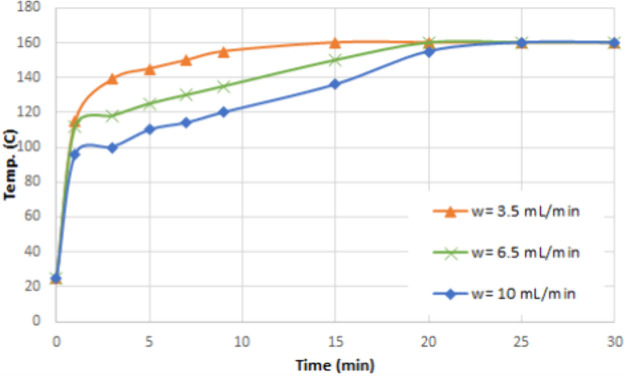
Dependence of the current temperature on time at different flow rates.

**Figure 4:**
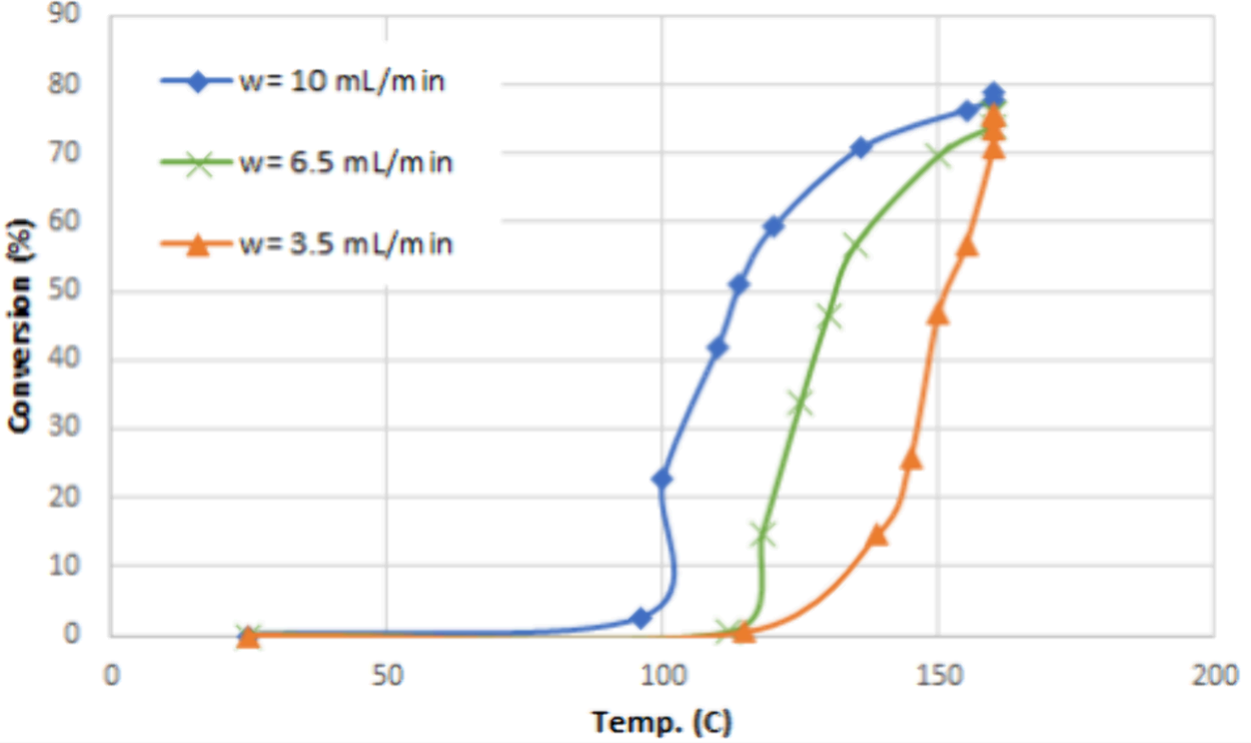
Dependence of the conversion on temperature at different flow rates.

The data pairs taken from Figures 2 and 3 allowed the construction of Figure 4.

It appears that up to ∼ 100 °C, within the investigated range (3.5–10 mL/min), the flow rate has no significant effect on the conversion rate (i.e., the curves hardly differ from one another), while the actual temperature plays a significant role. According to our measurements, an increase in the flow rate leads to lower reaction onset temperature. At a flow rate of 10 mL/min, the reaction already starts at 100 °C, and becomes significant at approximately 120 °C; whereas at 6.5 and 3.5 mL/min, the reaction begins only near 120 °C. Since our goal was to eliminate BPR, we could not exceed the boiling point of the reaction mixture (118 °C). Therefore, a volume flow rate of 10 mL/min proved to be a suitable starting point for our experiments.

#### 2.2.2 First Experiments

After eliminating the BPR from the system, we initiated a temperature profile from RT to 100 °C at a flow rate of 10 mL/min once the steady state had been reached. Even though the boiling point of butyl alcohol is 118 °C, we observed that the mixture boiled slightly above 90 °C. This caused the system to become unstable and uncontrollable (Figure 5, Exp. 1). Increasing the volume flow rate to 15 mL/min resulted in essentially the same behavior: the reaction proceeded slightly faster, but instability occurred earlier (Figure 5, Exp. 2). In the next experiment, we maintained the flow rate (15 mL/min) but reduced the temperature to 80 °C. This adjustment allowed us to achieve a stable operation, although the conversion rate increased more slowly (Figure 5, Exp. 3). Based on our previous experimental observations, it is proposed that the system “senses” the azeotropic boiling point of the mixture (93 °C), thereby preventing the reaction temperature of reaching 117 °C. Accordingly, we further increased the flow rate to 25 mL/min and set the temperature to 90 °C, which now appeared safe (Figure 5, Exp. 4).

**Figure 5:**
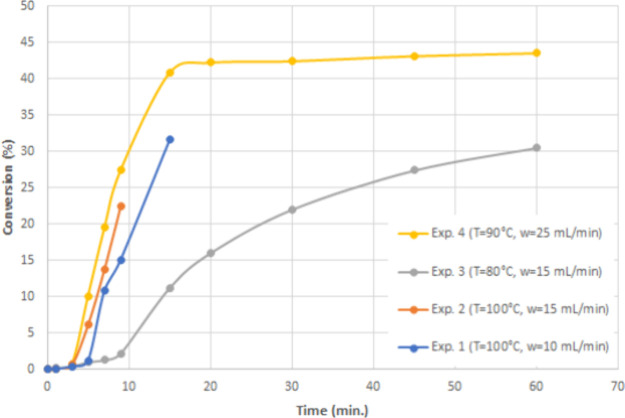
Dependence of the conversion on time at different flow rates and temperatures.

Comparing the outcome of the experiments, we found that the initial instability was avoided in Exp. 4, and under these conditions we achieved a maximum conversion of 44%, within 15 min. This behavior is consistent with our previous experience.

#### 2.2.3 Effect of Stirring

Given that the applied flow rates (w = 10–25 mL/min) are high relative to the reactor volume (V_f_= 10 mL), and considering that stirring is generally not applied in flow systems - that is consistent with high-flow technologies -, it is proposed that in the lack of stirring, the system operates under near plug-flow conditions. No stirring was applied in the next experiments.

Since our system is technically capable of stirring, we wished to explore whether it influences the reaction outcome. The experiment marked by Exp. Four was repeated under stirring (Exp. 5), and the results are shown in Figure 6.

**Figure 6:**
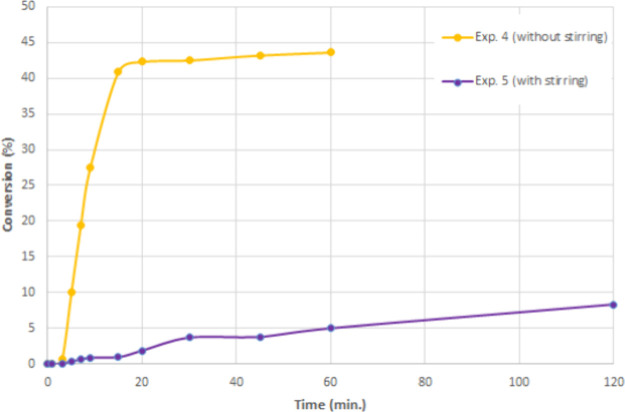
Dependence of the conversion on the stirring.

We were surprised to observe that, even after 2 h, the conversion under stirring did not reach 10%. We therefore sought an explanation for this unexpected phenomenon. First, it is important to note that the sensing point of the MW system’s infrared thermometer is positioned at the bottom to measure the temperature of the reaction mixture entering the reactor. Under plug-like flow conditions, the measured temperature is significantly lower than that in the upper regions of the reactor. Since the specific configuration of the apparatus did not allow for the integration of additional internal temperature probes or the relocation of the fixed IR sensor’s measurement point, a calibration experiment was carried out to assess the reliability of the IR temperature measurement under flow conditions. Butyl alcohol was used as a reference liquid with a known boiling point (118 °C). The system was operated under recirculating flow at different volume flow rates, and the IR-detected temperature was recorded at the onset of boiling.

The measurements were performed both under nonstirred and stirred conditions. It was found that the measured temperature systematically underestimated the actual temperature, and the deviation increased with increasing flow rate (Table 1). For example, at 10 mL/min the deviation was approximately 4 °C under stirred conditions, compared to about 55 °C in the absence of stirring; at 25 mL/min, these values increased to approximately 25 and 75 °C, respectively. This suggests that in the nonstirred system, the larger temperature discrepancy may give rise to pronounced local overheating effects.

**Table 1:** IR Sensor Readings at different Flow Rates at 118 °C Boiling Point: Stirring vs. No Stirring

	IR sensor temperature measured at boiling point (118 °C) [°C]	
flow rates [mL/min]	with stirring	without stirring
10	114	63
15	112	53
25	94	43

These results confirm that, under nonstirred flow conditions, the IR sensor does not accurately reflect to the bulk temperature of the reaction mixture. This supports the interpretation that temperature underestimation leads to MW power overcompensation and localized overheating. Since direct spatial temperature measurements (e.g., IR thermal imaging or insertion of internal probes) are not feasible in the applied commercial MW flow system due to geometrical and instrumental constraints. Therefore, the proposed explanation remains a hypothesis supported by indirect experimental evidence.

Without stirring, as a result of the linear flow pattern, a temperature gradient develops along the reactor length, from the inlet (bottom) toward the outlet (top). At the same time, under stirring, the heating induced by MW irradiation also affects the lower region, thereby preventing overheating in the upper zones (Figure 7).

**Figure 7:**
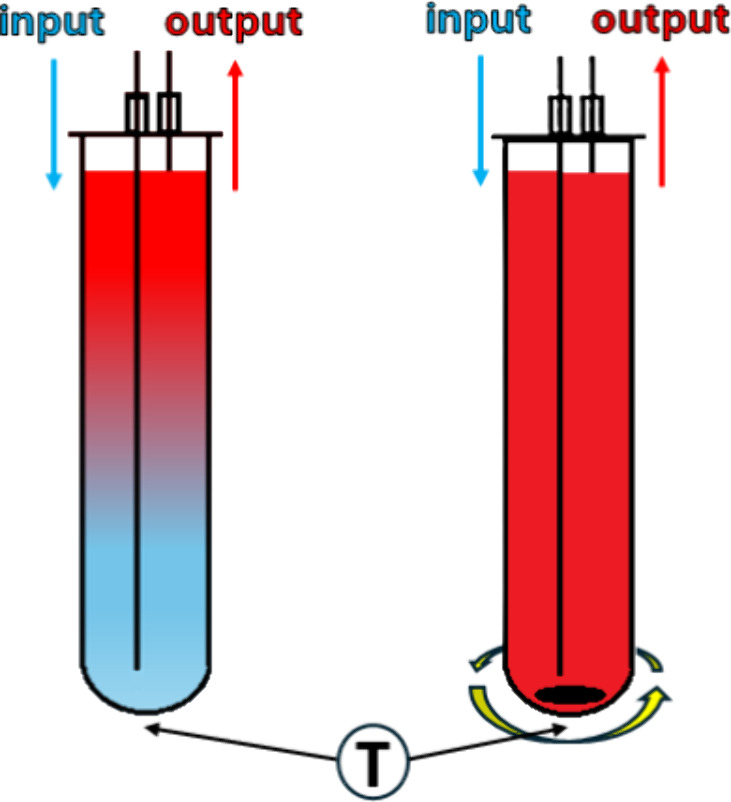
Schematic representation of the temperature profile in the reactor under conventional (nonstirred) flow conditions (left) and with stirring (right).

We observed that, from among the two reactions (Exp. Four and 5), the system operated under stirring showed higher stability. After optimizing the reaction parameters, we could maintain stable operation much easier, therefore, we also examined it from an energy perspective, as reported in our previous article. To facilitate the comparison, the time - energy diagrams for the two variations discussed above are shown in Figure 8.

**Figure 8:**
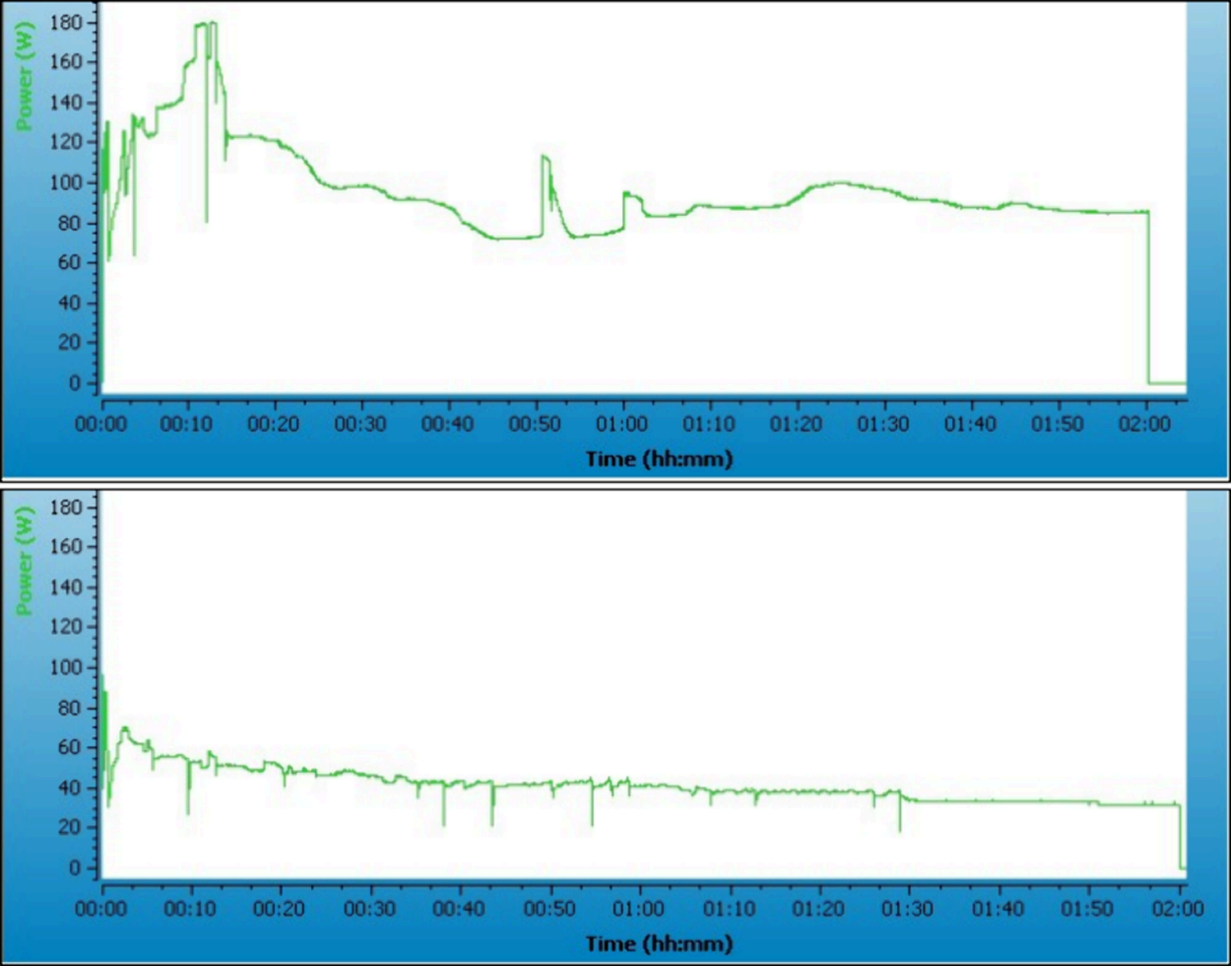
Energy requirement of the nonstirred (Exp. 4, top) and stirred (Exp. 5, bottom) reaction mixtures as a function of time.

In the case of a stirred reaction mixture, energy input is much more uniform, and the average of the energy uptake used is significantly lower (45 W vs 100 W). The maximum instantaneous energy value is also considerably lower (90 W vs 180 W), however, under these conditions, the reaction tends not to proceed as expected. This comparison further supports the fact that, in a stirred reaction mixture, the energy input primarily serves to maintain the desired temperature. To quantify the difference, we calculated and compared the previously introduced E_t_.^23^ This represents the amount of energy consumed to reach 90% of the maximum achievable conversion. By subtracting the energy required to maintain the system in the absence of reaction (E_b_), the energy specifically needed for the reaction: E_r_ [Wh] = E_t_-E_b_ may be obtained. For the unstirred system, E_r_ (Exp. 4) = 22 Wh; whereas for the stirred system, E_r_ (Exp. 5) = 26 Wh. Considering the difficulty of precise integration and the fact that, under stirring, the system must maintain its temperature for a longer period to reach 90% of the maximum conversion available in a given reaction, the two values are very close. Calculating the average power requirement from the above values yields the following results: P̅ _r_ (Exp. 4) = 33 W and P̅ _r_ (Exp. 5) = 19 W. Although the total energy input over the course of the process is similar, the nonstirred system operates at a much higher power demand. This means that the excitation is more efficient, resulting in a faster reaction. For clarity, the difference is illustrated in Figure 9.

**Figure 9:**
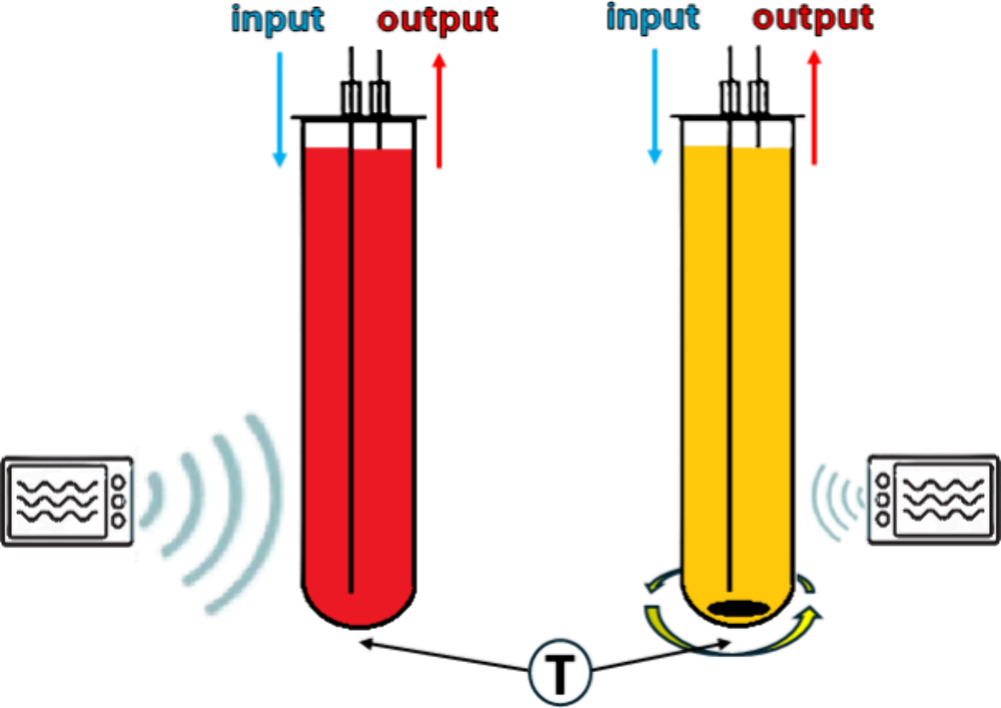
Average power of the magnetron acting on the reaction mixture is shown on the left for ’classical’ flow accomplishment (left) vs under stirring (right).

When stirring is applied, the medium entering the reactor is instantaneously mixed, and the observed temperature increase partly reflects homogenization rather than solely higher MW power input. This homogenization eliminates local temperature gradients within the reactor.

In contrast, under nonstirred conditions, the IR sensor underestimates the temperature at the inlet, leading to increased microwave power input and the formation of localized overheating zones along the reactor.

Although the total energy input is comparable in both cases, its spatial and temporal distribution differs significantly. This suggests that localized high-temperature regions may play a key role in driving the reaction under nonstirred conditions.

While nonthermal microwave effects cannot be excluded, the observed behavior can be rationalized by thermal heterogeneity within the reactor.

The possibility of ionic liquid depletion from the active microwave zone under stirring was also considered, but the significant temperature gradient observed in the nonstirred system remains the most plausible explanation.

#### 2.2.4 Final Optimization of the Flow System: Handling the Problem of the Azeotrope Formed

In the previous experimental series, the negative effect of boiling point depression associated with the formation of the azeotrope, as well as the role of stirring, were observed. In the following experiments, the effects of these two parameters were further investigated. Our aim was to remove the water generated during the reaction using drying agents (Na_2_SO_4_, MgSO_4_ and molecular sieves); and therefore, the existing MW setup was modified as shown in Figure 10.

**Figure 10:**
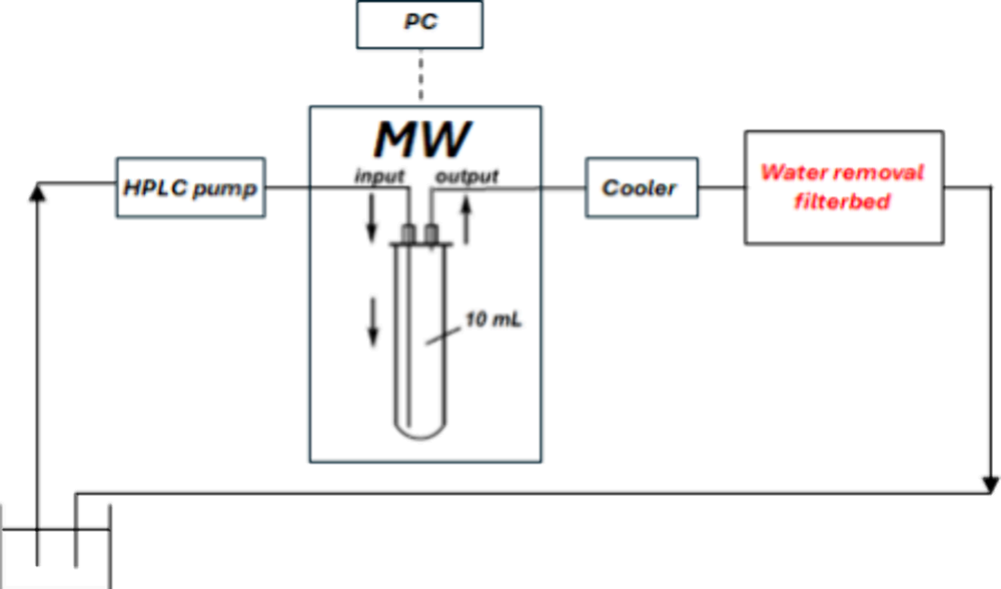
Recirculating MW system modified with a water-removing filter bed.

The drying agent was placed in a cartridge equipped with a filter at the outlet, through which the reaction mixture was circulated. The water formed was removed via the minimum-boiling azeotrope allowing the reaction temperature to be increased. In the first series of experiments, the effect of Na_2_SO_4_ was investigated both with and without stirring. The previously optimized flow rate of 25 mL/min was maintained, and the results were compared with those obtained in Exp. Four and Exp. Five (Figure 11).

**Figure 11:**
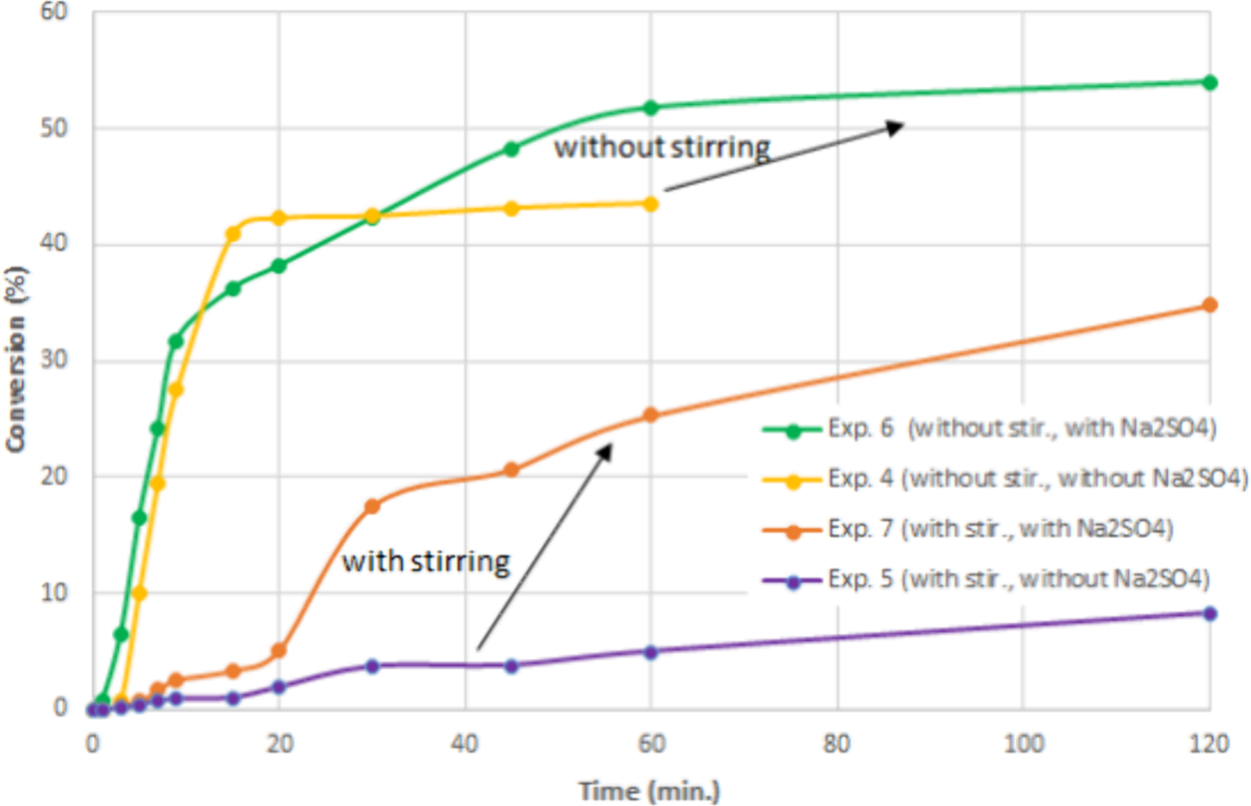
Effect of Na_2_SO_4_ (Exp. Six vs Exp. 4) and the combined effect of Na_2_SO_4_ and stirring (Exp. Seven vs Exp. 5) on conversion as a function of time.

The use of Na_2_SO_4_ as a drying agent influenced positively the course of the reaction in both cases. After the water was removed, the reaction temperature could be increased to the desired 117 °C. The presence of the drying agent increased the final conversion by approximately 10% in the unstirred experiment, and by more than 25% under stirred conditions.

The previous experiment was repeated using molecular sieves (Figure 12, Exp. 8). Based on a series of measurements, we observed that reducing the flow rate improved the stability of the system; therefore, despite the fact that the lower flow rates reduced the conversion in batch experiments, this approach was investigated for both Na_2_SO_4_ (Figure 12, Exp. 9) and MgSO_4_ (Figure 12, Exp. 10) . As MgSO_4_ performed slightly better than Na_2_SO_4_, the effect of classical acid catalysis was further examined using p-toluenesulfonic acid (pTsOH) in the presence of MgSO_4_ (Figure 12, Exp. 11) . The data obtained are presented in Figure 12, which also includes the reference data from Exp. 6.

**Figure 12:**
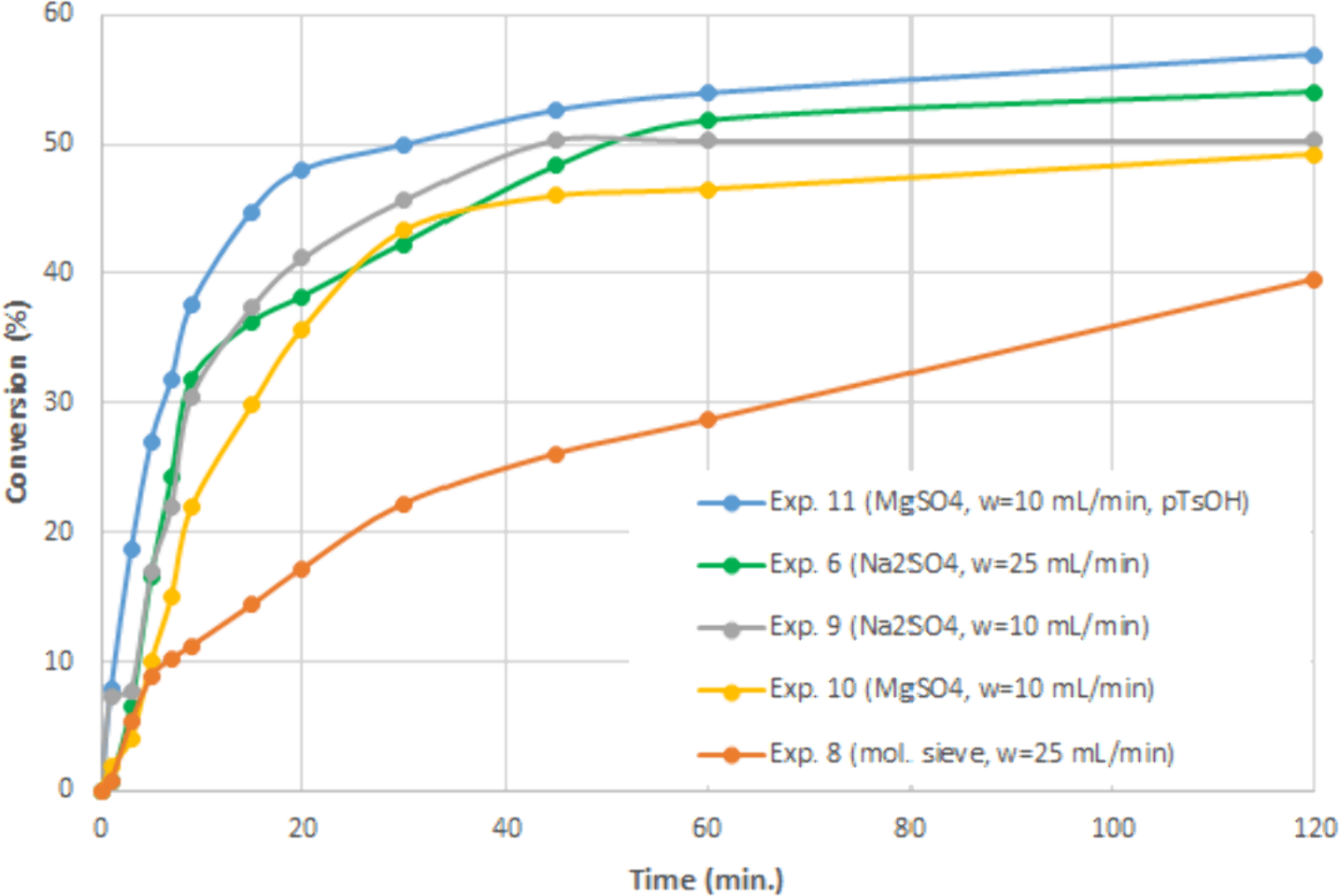
Effect of the drying agents on the conversion as a function of time.

Among the investigated drying agents, molecular sieves were the least effective, followed by Na_2_SO_4_ and then MgSO_4_. It is proposed that, despite their inherently high-water adsorption capacity, the performance of molecular sieves is strongly limited under the applied conditions. In the present setup, the drying agent is placed in an external cartridge and operated under relatively high flow rates, resulting in short residence times and limited mass transfer.

Under these conditions, the diffusion of water molecules into the internal pore structure of the molecular sieves is restricted, and equilibrium adsorption cannot be established within the time scale of the process. Consequently, molecular sieves cannot effectively compete with the faster-acting inorganic drying agents.

The fastest reaction rate and, consequently, the highest conversion, were achieved in the presence of MgSO_4_ combined with the pTsOH catalyst.

All measurements were repeated at least once, and the data could be reproduced within a standard deviation ± 2%.

In summary, our key findings are the following:

- Operating without the BPR allows for atmospheric pressure conditions reducing safety risks.
- Without stirring, the high flow rates (10–25 mL/min) relative to the reactor volume (10 mL) result in plug-like flow.
- Operation without stirring led to the instability of the system due to inaccurate temperature measurement by the IR sensor causing an increased energy input. Although the nonstirred system is technologically unstable, the overcompensation results in a higher conversion and an efficient reaction. In contrast, when the mixture is stirred, the system becomes stable, but only low conversion occurs.
- To address the problem, a drying agent (Na_2_SO_4_, MgSO_4_, or molecular sieves) was used to remove the water formed, eliminating the azeotrope with a minimum-boiling point, which otherwise limited the maximum applicable temperature.
- MgSO_4_ allowed for the fastest reaction rate and the highest conversion.

## 3 Conclusions

In this study, we aimed at eliminating the back-pressure regulator (BPR) from the flow system to simplify the setup, to make it more robust and improve operational safety. Feasibility tests showed that precise temperature control is critical. It was found that in the CEM MW reactor applied in a flow mode, the built-in IR temperature sensor provides inaccurate values in the absence of stirring, leading to an excessive energy input. This problem does not occur in batch reactions due to the uniform temperature distribution ensured by stirring, however, the issue arises when the batch reactor is replaced by the manufacturer’s professional flow cell, where the temperature measurement occurs at bottom, where the system inlet is placed. The introduction of the room temperature medium causes overcompensation, and results in unstable flow operation.

Although stirring stabilizes the system, the reaction then proceeds only in lower conversions. Further complications arise from the formation of the binary azeotrope with a minimum-boiling point containing butyl alcohol and the water formed during esterification, which limits the applicable temperature range. Removal of the water with inexpensive drying agents, and reduction of the flow rate stabilized successfully the flow system allowing a reliable operation.

The presented approach may contribute to the safer implementation of MW-assisted flow processes in laboratory and pilot-scale applications, allowing robust operation at atmospheric pressure.

The results and the modified technological setup enable the methodology to be extended to other model systems, with the ultimate goal of establishing a stable and productive chemical-technological platform capable of handling various PFEx or SuFEx hubs.

## Supplementary Material



## Notes

Experimental details and general procedures for the microwave-assisted recirculating flow esterification experiments (PDF)

## Abbreviations

BPR: back pressure regulator
MW: microwave
IL: ionic liquid
