# Supplementary material for: Practical Microwave-Assisted Recirculated Flow Esterification at Atmospheric Pressure
Source: ACS Omega. 2026 Jun 2;11(23):34636–43. doi: 10.1021/acsomega.6c03090 (PMC13281024; doi:10.1021/acsomega.6c03090)
Supplement: Supplementary file 1 [file ao6c03090_si_001.pdf]

# A Practical Microwave-assisted Recirculated Flow Esterification at Atmospheric Pressure

*József Schindler*, Rebeka Harján and György Keglevich*

*Department of Organic Chemistry and Technology, Faculty of Chemical Technology and Biotechnology, Budapest University of Technology and Economics, 1111 Budapest, Műegyetem rkp. 3, Hungary. E-mail:** schindler.jozsef@vbk.bme.hu†

## S1 General Information.

The MW reactions were carried out in a CEM Discover (300 W) focused microwave reactor (CEM Microwave Technology Ltd., Buckingham, U.K.) equipped with a 10 mL flow cell available from the supplier of CEM using 40−200 W irradiation under isothermal conditions. The reaction temperature was monitored by an external IR sensor. LC–MS measurements were performed with an Agilent 1200 liquid chromatography system, coupled with a 6130 quadrupole mass spectrometer equipped with an ESI ion source (Agilent Technologies, Palo Alto, CA, USA).

## S2 Preparation of the reaction mixture

A mixture of 10 g (70.4 mmol) $Ph_2P(O)H(OH)$ and 1.0 g (3.5 mmol) of [bmim][PF6] and 100 mL (1.1 mol) of butyl alcohol was homogenized by stirring at 25˚C for 15 min.

## S3 General procedure for the circulated flow esterification of phenyl-H-phosphinic acid (1) with butyl alcohol

A 25 mL mixture containing PhP(O)H(OH) (1) and 5% of [bmim][PF6] and nBuOH in a ratio of 1: 15.6 was circulated applying the corresponding rate at 25˚C. After the mixture filled the system, the flow cell was irradiated with a power of 300 W until the desired temperature was reached. After this, the power was controlled automatically (by 40–200 W) to maintain the value set. The operation was regarded steady state after 20–120 min reaching the "plato" as suggested by LC-MS analysis.

A part of the measurements was repeated, and the data could be reproduced within a standard deviation ± 2%.
